# Proposers’ Economic Status Affects Behavioral and Neural Responses to Unfairness

**DOI:** 10.3389/fpsyg.2017.00847

**Published:** 2017-05-29

**Authors:** Yijie Zheng, Xuemei Cheng, Jialin Xu, Li Zheng, Lin Li, Guang Yang, Xiuyan Guo

**Affiliations:** ^1^Shanghai Key Laboratory of Magnetic Resonance, East China Normal UniversityShanghai, China; ^2^Department of Physics, East China Normal UniversityShanghai, China; ^3^School of Psychology and Cognitive Science, East China Normal UniversityShanghai, China; ^4^Shanghai Key Laboratory of Brain Functional Genomics, Key Laboratory of Brain Functional Genomics, Ministry of Education, East China Normal UniversityShanghai, China; ^5^National Demonstration Center for Experimental Psychology Education, East China Normal UniversityShanghai, China

**Keywords:** ultimatum game (UG), economic status, fMRI, perception of unfairness, decision making

## Abstract

Economic status played an important role in the modulation of economic decision making. The present fMRI study aimed at investigating how economic status modulated behavioral and neural responses to unfairness in a modified Ultimatum Game (UG). During scanning, participants played as responders in the UG, and they were informed of the economic status of proposers before receiving offers. At the behavioral level, higher rejection rates and lower fairness ratings were revealed when proposers were in high economic status than in low economic status. Besides, the most time-consuming decisions tended to occur at lower unfairness level when the proposers were in high (relative to low) economic status. At the neural level, stronger activation of left thalamus was revealed when fair offers were proposed by proposers in high rather than in low economic status. Greater activation of right medial prefrontal cortex was revealed during acceptance to unfair offers in high economic status condition rather than in low economic status condition. Taken together, these findings shed light on the significance of proposers’ economic status in responders’ social decision making in UG.

## Introduction

Human behaviors in social decision-making are under the influence of unfairness-related decision making. In the past decades, an abundance of findings were provided that people insisted on maintaining fairness norms even at the cost of themselves. Among all the economic games, Ultimatum Game (UG) is a primary experimental tool used to explore the underlying mechanisms of human fairness (Guth et al., 1982; Thaler, 1988; Camerer and Thaler, 1995). A typical UG involves two players, one player (proposer) decides how to split a sum of money, and the other one (responder) decides whether to accept the division or not. If the responder accepts, both of them get the suggested division of money, otherwise they received nothing. Past researches revealed that, in spite of personal loss, people would reject extremely unfair offers to punish norm-violating behaviors (Guth et al., 1982), indicating the importance of perception of unfairness in social decision making.

Several fairness-related brain regions involved in UG, such as anterior insula (AI), anterior cingulate cortex (ACC) and dorsolateral prefrontal cortex (DLPFC), have been identified in previous neuroimaging studies (Sanfey et al., 2003; Guroglu et al., 2010, 2011). It was suggested that the involvement of AI and ACC in UG were associated with negative affect elicited by unfair offers and with detecting and responding to violating fairness-related norms (Sanfey et al., 2003; Montague and Lohrenz, 2007; Guroglu et al., 2010, 2011). Moreover, the activation of DLPFC was associated with top–down inhibition of self-interested impulses to accept unfair offers and with integrating information and selecting appropriate responses to unfair offers (Buckholtz et al., 2008; Guroglu et al., 2010; Buckholtz and Marois, 2012).

Previous researches have demonstrated that economic status played a role in the modulation of economic decision making (Holm and Engseld, 2005; Haile et al., 2008). It was indicated that during a UG task, proposers preferred to give higher offers to responders in low economic status rather than ones in high economic status (Holm and Engseld, 2005). It was also revealed that with the increase of age, children began to take others’ economic status into account during resource allocation, i.e., they would give more resource to poor individuals than wealthy ones during a resource allocation task (Paulus, 2014).

However, most studies focused on how proposers might consider economic status of responders in the bargaining, few of them shed light on the effect of proposers’ economic status during responders’ decision making. Previous research demonstrated that people in the higher socioeconomic status who owned more resources were likely to engage in more prosocial behaviors and volunteered more (Granzin and Olsen, 1991; Penner et al., 2005; Piff et al., 2010) than others who were in the lower socioeconomic status. Thus individuals in superior economic status might be expected to give a higher offer in the UG task and therefore unfair offers proposed by high socioeconomic status individuals might induce larger discrepancy with expectation, resulting in stronger emotional response. Hence it is likely that people would reject more unfair offers from people in superior economic status.

In the present study, we used a modified UG in which participants acted as responders and were informed of proposers’ economic status before receiving offers. We aimed to investigate three following questions. Firstly, we were interested in how proposers’ economic status modulated responders’ perception of unfairness. It was predicted that participants would consider proposers’ economic status so that they would feel higher level of unfairness when offered by proposers in superior economic status rather than those in inferior status. Secondly, we tried to reveal how proposers’ economic status modulated participants’ response to unfair offers. We predicted that participants would reject more unfair offers from proposers in superior (relative to inferior) economic status. Moreover, we tried to explore the neural mechanisms underlying the modulating effect of proposer’s economic status on unfairness-related social decision making. Recent studies have shown that thalamus (Zink et al., 2008; Hu et al., 2016), which was associated with social emotional arousal, and the medial prefrontal cortex (MPFC) (Zink et al., 2008; Wang et al., 2014), which was related to recognizing intentions and motives of others, were involved in processing of social status. As social status and economic status are both included in social hierarchies (Zink et al., 2008), the thalamus and MPFC may also engaged in encode economic status by processing responders’ perception of unfairness and responses to unfair offers related to economic status.

## Materials and Methods

### Participants

Twenty one right-handed volunteers [12 females, mean age = 22.8 ± 1.4 (SD) years] took part in this study. All the participants had normal or corrected-to-normal vision and none of them reported any abnormal neurological history. One participant was excluded from further statistical analyses due to severe head motion (>3°) during scanning. And two participants had to be excluded because of no acceptance responses to unfair offers in the *High* economic status condition. Written informed consent was acquired from all the participants. The study was approved by the Ethics Committee of East China Normal University.

### Materials

Seventy two common Chinese names were abbreviated (i.e., “Zhang L.” was an abbreviation of “Zhang Liang”) and displayed as proposers in the procedure. These names were randomly allocated to two conditions (*Economic Status*: *High* and *Low*). In each condition, there were 36 names [12 for fair proposals (¥25: ¥25), and 6 for each of unfair proposals (¥5: ¥45, ¥10: ¥40, ¥15: ¥35 and ¥20: ¥30)]. The gender and number of words were counterbalanced across names in different conditions.

### Procedure

**FIGURE 1:**
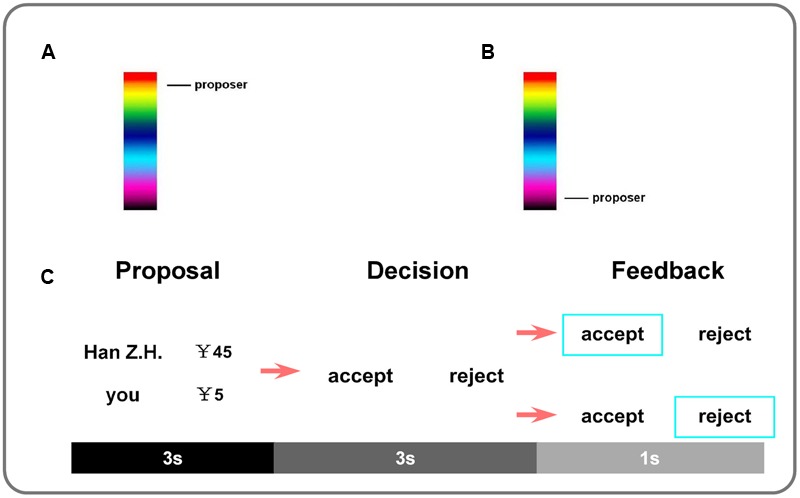
**Participants were scanned while playing the game for 12 blocks (six High economic status blocks and six Low economic status blocks).** Proposer’s economic status was displayed at the beginning of every block. **(A)** An arrow pointing to the top of the histogram indicating the *High* economic status, **(B)** An arrow pointing to the bottom of the histogram indicating the *Low* economic status. In each block, there were two fair proposals and four different unfair proposals (¥5: ¥45, ¥10: ¥40, ¥15: ¥35 and ¥20: ¥30). **(C)** In each trial, the proposal screen was presented for 3 s to display the split between the proposer and the participant (responder). Then the decision cue appeared and participants were required to decide whether to accept or reject the offer within 3 s by pressing corresponding buttons. Once they responded, a blue frame outside the selected choice would be presented for 1 s to provide feedback of their decision.

Participants were told that they would participate in an economic game with 72 different partners (students from the same university with them), along with an instruction introducing the rule of the game. Participants were told that proposers’ monthly family income was collected as economic status using a 10-step economic ladder in this study (Adler et al., 2000), with step-1 indicating the lowest economic status, and step-10 indicating the highest economic status. The economic status of the proposers in this study was either on step-1 or on step-10. They were also told that the proposals were obtained from different proposers before the experiment. And participants would also be asked about their own economic status after the experiment (**Figure 1**). Then participants were told that they would be presented with a proposal from one of the proposers about how to split ¥50 between them, and they could decide to either accept or reject the proposal with acceptance leading to the suggested split and rejection leaving both of them nothing. As for the payment, participants were told that both of themselves and the proposers would be paid according to their decision in each trial after some kind of transformation. They would be paid with a basic payment for their participation (¥50, ≈8.04 US$) plus the amount of money obtained from a random selection of 6% trials in the game.

Before scanning, participants practiced four blocks included 24 trials on a laptop. There were 12 blocks during scanning, including six blocks in which all the proposers were in high economic status and another six blocks in which all the proposers were in low economic status. Different status blocks were alternated with one another and counterbalanced across the participants (ABABABABABAB for half of the participants and BABABABABABA for the rest). Each block lasted for 70∼75.8 s, with a 5-s rest between every two blocks. Before each block, a 6-s cue of proposer’s economic status was displayed by a graduated color histogram to inform the participants about the economic status of the proposers in the following block, with an arrow pointing to the bottom of the histogram indicating the *Low* economic status, and pointing to the top of the histogram indicating the *High* economic status. Each block contained two fair proposals and four different unfair proposals (¥5: ¥45, ¥10: ¥40, ¥15: ¥35, and ¥20: ¥30). All of the trials in a block were presented in a random order. For each trial, the proposal screen was presented for 3 s to display the split between the proposer and the participant (responder). Then a decision cue appeared and participants were required to decide whether to accept or reject the offer within 3 s by pressing the corresponding buttons of the magnet-compatible button Box (i.e., right index finger for acceptance and right middle finger for rejection). Once they responded, a blue frame outside the selected choice would be presented for 1 s to provide participants with the feedback of their decision. The intervals between trials were jittered from 2 to 4 s. There was also one jittered blank (500∼1500 ms) between the proposal screen and the decision cue.

After scanning, the same stimuli including proposers’ economic statuses and proposals were presented again. Participants were asked to rate the fairness of each offer on a 9-point Likert-type scale with 1 indicating extremely unfair and 9 indicating extremely fair. And participants were also asked to rate their own economic status from 1 indicating the lowest economic status and 10 indicating the highest economic status.

### fMRI Image Acquisition and Analysis

Participants were scanned using a 3T Siemens scanner at the Shanghai Key Laboratory of Magnetic Resonance of East China Normal University. Firstly, we acquired anatomical images of each participants using a T1-weighted, multiplanar reconstruction (MPR) sequence (TR = 2530 ms, TE = 2.34 ms, 192 slices, slice thickness = 1 mm, FOV = 256 mm, matrix size = 256 ∗ 256) (Cheng et al., 2015; Wang et al., 2015). After that, a gradient-echo echo-planar imaging (EPI) was used to acquire the sequence functional images (TR = 2400 ms, TE = 30 ms, FOV = 220 mm, matrix size = 64 ∗ 64, 39 slices, slice thickness = 3 mm) (Cheng et al., 2015; Wang et al., 2015).

SPM8 software package (Wellcome Department of Cognitive Neurology, London) was employed to perform the preprocessing and statistical analyses of brain imaging data. The first five functional images were excluded from each subject to allow scanner equilibrium effects. Then, all functional images were slice timing corrected, realigned, normalized into the MNI space (resampled at 2 mm ∗ 2 mm ∗ 2 mm voxels), and smoothed with an 8-mm full-width half maximum isotropic Gaussian kernel (Cheng et al., 2015).

First-level analyses were then performed for each subject using general linear models (GLM) implemented in SPM8. We modeled onsets of the proposal screens and onsets of the decision cues for six types of events, including *LF* (fair offers in the *Low* economic status condition), *LUA* (accepted unfair offers in the *Low* economic status condition), *LUR* (rejected unfair offers in the *Low* economic status condition), *HF* (fair offers in the *High* economic status condition), *HUA* (accepted unfair offers in the *High* economic status condition) and *HUR* (rejected unfair offers in the *High* economic status condition). Additionally regressors of no interest were the cues of proposers’ economic status, the feedbacks for acceptance, the feedbacks for rejection, and proposal screen and decision cue for trials which participants failed to respond to. All these regressors were modeled with zero duration and convolved with a canonical hemodynamic response function (HRF). Moreover, six realignment parameters and one overall mean during the whole phase were included in the design matrix as well. To filter the low-frequency noise, a cutoff of 192 s was applied. During first-level analyses, six contrast images (*LF, LUA, LUR, HF, HUA, HUR*) for proposal presentation were acquired from each participant and were fed into another flexible design in the second-level analyses.

Brain activities related to unfairness were defined by contrasting fair trials with unfair trials and the reverse contrasts. Brain activations corresponding to economic status were identified by the (*High* – *Low*) and reverse contrasts. The (Reject–Accept)_Unfair_ and reverse contrasts were tested to compute brain activations related to participant’s responses (rejecting and accepting unfair offers). Then, the economic status ∗ unfairness interactions defined by (Unfair – Fair)_High_ – (Unfair – Fair)_Low_ and their reverse contrasts were computed to explore how contexts affect unfairness in all trials. The economic status ∗ response interactions defined by (Reject – Accept)_Unfair High_ – (Reject – Accept)_Unfair Low_ and their reverse contrasts were also tested to extract specific regions showing modulation of responders’ responses to unfair offers by different contexts. A voxel-level threshold of *p* < 0.001 (uncorrected) and a cluster-level FWE correction *p* < 0.05 were used. To further test how the economic status affected brain activations to perception of unfairness and response to unfair offers, specific activations identified in the interactions were used to compute regions of interest (ROIs). All the significant voxels in the activated clusters within 6 mm spherical regions centered on the peak or local maximum coordinates were included in each ROI. Beta estimates across ROIs were extracted for further statistics using the MarsBaR toolbox in SPM8.

## Results

### Behavioral Results

#### Fairness Ratings

**FIGURE 2:**
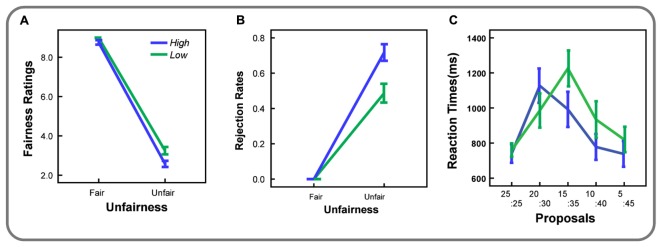
**(A)** Fairness ratings, **(B)** Rejection rates, and **(C)** Reaction times were plotted as a function of unfairness level in both economic status conditions. Error bars indicate SEM.

The behavioral variable of interest was the fairness ratings of UG offers (**Figure 2A**). A 2 (economic status: low vs. high) ∗ 2 (unfairness: fair vs. unfair) ANOVA revealed significant main effects of economic status [*F*(1,17) = 28.00, *p* < 0.01, $\eta_{p}^{2}$ = 0.62) and unfairness [*F*(1,17) = 1575.88, *p* < 0.01, $\eta_{p}^{2}$ = 0.99], indicating higher ratings in the *Low* economic status condition than in the *Hig*h economic status condition and decreased ratings to unfair offers than fair offers. The interaction was significant [*F*(1,17) = 16.42, *p* < 0.01, $\eta_{p}^{2}$ = 0.49]. Paired *t*-tests revealed higher ratings in the *Low* economic status condition relative to the *High* economic status condition whether the offers were fair [*t*(17) = 7.42, *p* < 0.01, Cohen’s *d* = 0.50] or not [*t*(17) = 2.12, *p* < 0.05, Cohen’s *d* = 1.75].

#### Rejection Rates and Reaction Times (RT)

The behavioral variable of interest was the rejection rates (**Figure 2B**). A 2 (economic status: low vs. high) ∗ 2 (unfairness: fair vs. unfair) repeated-measures analysis of variance (ANOVA) revealed significant main effects of economic status [*F*(1,17) = 118.96, *p* < 0.01, $\eta_{p}^{2}$ = 0.88] and unfairness [*F*(1,17) = 622.42, *p* < 0.01, $\eta_{p}^{2}$ = 0.97], indicating higher rejection rates in high economic status than in low economic status conditions. A significant interaction was also found [*F*(1,17) = 118.96, *p* < 0.01, $\eta_{p}^{2}$ = 0.88]. Further paired *t*-tests showed that, although participants accepted all the fair offers, they rejected some of the unfair offers. Rejection rates for unfair trials in the *High* economic status condition were significantly higher than those in the *Low* economic status condition [*t*(17*)* > 10.91, *p* < 0.01, Cohen’s *d* = 2.57].

Given that UG is a time-consuming social decision making task, which involves complicated trade-off among motivations favoring either acceptance or rejection, reaction times (RT) was also analyzed in the present study. For RTs, a 2 (economic status: low vs. high) ∗ 5 (unfairness level: ¥25: ¥25 vs. ¥20: ¥30 vs. ¥15: ¥35 vs. ¥10: ¥40 vs. ¥5: ¥45) ANOVA was also carried out (**Figure 2C**). Results showed the main effects of economic status [*F*(4,68) = 5.48, *p* < 0.05, $\eta_{p}^{2}$ = 0.24) and unfairness level [*F*(4,68) = 17.70, *p* < 0.01, $\eta_{p}^{2}$ = 0.51], indicating longer RTs in the *Low* economic status condition than in the *High* economic status condition, and longer RTs for medium unfairness levels (¥20: ¥30 and ¥15: ¥35) than for high unfairness levels (¥10: ¥40 and ¥5: ¥45). The interaction was also significant [*F*(4,68) = 6.43, *p* < 0.01, $\eta_{p}^{2}$ = 0.27]. Paired *t*-tests revealed that, RTs for trials of ¥15: ¥35 in the *Low* economic status condition were significantly longer than RTs for trials of other offers (*ts* > 3.55, *ps* < 0.01, Cohen’s *ds* > 0.83). And in the *High* economic status condition, the average of RTs for trials of ¥20: ¥30 was the longest among all offer conditions. RTs for trials of ¥20: ¥30 were significantly longer than that of other offers (*ts* > 5.62, *ps* < 0.01, Cohen’s *ds* > 1.32), except for RTs for trials of ¥15: ¥35 [*t*(17) = 1.64, *p* > 0.1]. We then carried out a further chi-square test to investigate whether the probability of observing longest RT from a participant on two unfairness levels (¥20: ¥30, ¥15: ¥35) would be different between economic statuses. Results revealed significant difference when proposers were in high and low economic status (χ^2^ = 7.20, *p* < 0.05, *w* = 1.07).

Additionally, a one-sample *t*-test was also used to check participants’ own economic status. The average rating was 4.67 ± 0.97 (SD), which was significantly higher than the *Low* economic status and lower than the *High* economic status (*ts* > 16.03, *ps* < 0.01, Cohen’s *d* = 3.78). It indicated that economic status of participants was inferior to the *High* economic status and superior to the *Low* economic status.

### fMRI Results

#### Unfairness-Related Effects: Economic Status × Unfairness Interaction

**FIGURE 3:**
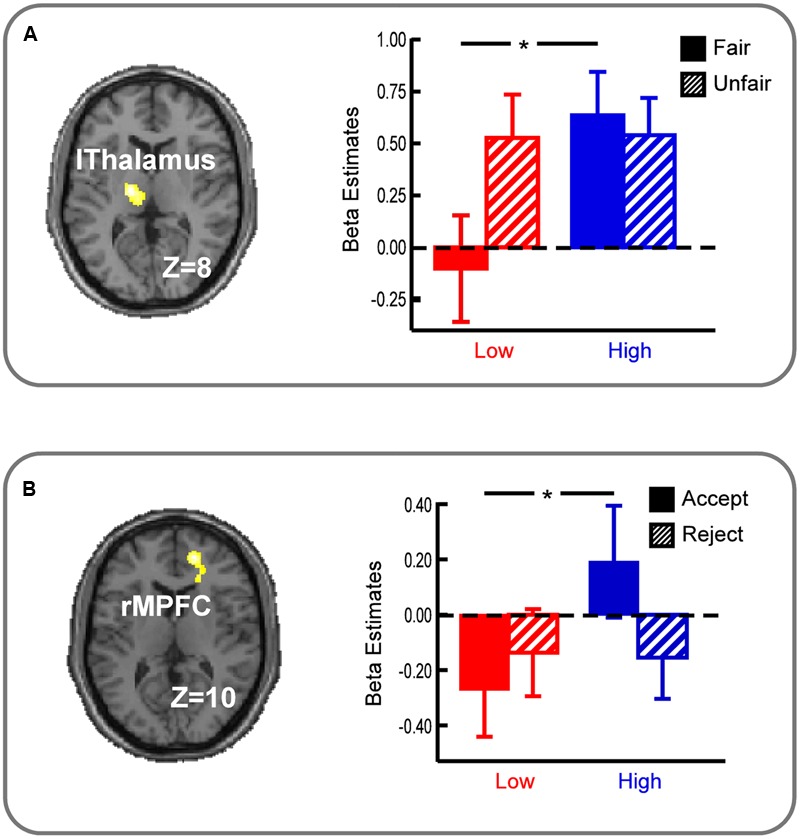
**(A)** Left Thalamus showed the modulation of economic status on perception of unfairness. **(B)** Right MPFC showed the modulation of economic status on responses to unfair offers. l, left hemisphere; r, right hemisphere. All the activations survived the voxel-level threshold of uncorrected *p* < 0.001 with the cluster-level threshold of Family wise error (FWE) corrected *p* < 0.05. Error bars indicate SEM. ^∗^*p* < 0.01.

Interaction between unfairness and economic status was computed by the [(Unfair – Fair)_Low_ – (Unfair – Fair)_High_] and the reverse contrasts. The [(Unfair – Fair)_Low_ – (Unfair – Fair)_High_] contrast showed activations in left thalamus (MNI -16 -14 8), and the reverse contrast showed no significant activations (**Table 1**). As shown in **Figure 3A**, analyses on beta estimates revealed that left thalamus activated stronger in the *HF* conditions compared with the *LF* conditions (*p* < 0.01), and showed no significant activation difference between the *HU* conditions and the *LU* conditions (*p* > 0.1).

**Table 1:** Regions showing unfairness ^∗^ economic status interactions and responses during unfair trials ^∗^ economic status interactions.

		Peak activation				
	Region	*X*	*Y*	*Z*	*t*-Value	Voxels
**(Unfair – Fair)_Low_ – (Unfair – Fair)_High_**						
L	Thalamus	-16	-14	8	4.66	215
**(Unfair – Fair)_High_ – (Unfair – Fair)_Low_**						
	No regions					
**(Reject – Accept)_Unfair Low_ – (Reject – Accept)_Unfair High_**						
R	MPFC	16	50	10	5.16	253
**(Reject – Accept)_Unfair High_ – (Reject – Accept)_Unfair Low_**						
R	Precuneus	22	-58	32	5.17	271
R	Cerebellum	14	-64	-18	5.01	238

*Coordinates (mm) are in MNI space. L, left hemisphere; R, right hemisphere. *p* < 0.001 (uncorrected), cluster-level FWE correction *p* < 0.05.*

Additionally, main effect of unfairness was also computed by the (Unfair – Fair) and the reverse contrasts. Consistent with previous researches (Sanfey et al., 2003; Guroglu et al., 2010, 2011), the (Unfair – Fair) contrast revealed activations in bilateral DLPFC (MNI -48 26 32; 40 16 42), left AI (MNI -30 20 4) and right ACC (MNI 10 34 28). No region of interest was activated in the reverse contrast (**Table 2**).

**Table 2:** Regions showing main effects of unfairness.

		Peak activation				
	Region	*X*	*Y*	*Z*	*t*-Value	Voxels
**Unfair – Fair**						
R	Putamen	16	14	0	8.20	1032
L	Inferior parietal gyrus	-30	-56	44	8.11	6118
R	Superior medial frontal gyrus	4	24	44	7.55	1822
R	ACC	10	34	28	5.26	
L	AI	-30	20	4	6.70	747
R	DLPFC	40	16	42	6.00	1570
L	Middle frontal gyrus	-28	2	56	5.86	278
R	Inferior occipital gyrus	44	-78	-2	5.31	481
L	DLPFC	-48	26	32	5.10	1081
**Fair – Unfair**						
R	Supramarginal gyrus	64	-26	28	4.94	299

*Coordinates (mm) are in MNI space. L, left hemisphere; R, right hemisphere. *p* < 0.001 (uncorrected), cluster-level FWE correction *p* < 0.05.*

#### Response-Related Effects during Unfair Trials: Economic Status × Response Interaction

Interaction between responses and economic status was computed by the [(Reject – Accept)_Unfair Low_ – (Reject – Accept) _Unfair High_] and the reverse contrasts. The [(Reject – Accept) _Unfair Low_ – (Reject – Accept)_Unfair High_] contrast showed activations in right MPFC (MNI 16 50 10), and no region of interest was activated in the reverse contrast (**Table 1**). Right MPFC revealed to be more active in the *HUA* conditions than in the *LUA* conditions (*p* < 0.01), and showed no significant activation difference between the *HUR* conditions and the *LUR* conditions (*p* > 0.1) (**Figure 3B**).

## Discussion

In this study, we used a modified UG to investigate how economic status of proposers modulated responders’ unfairness-related decision making and the underlying neural mechanisms. When facing with proposers in high (relative to low) economic status, participants felt higher level of unfairness, and significant left thalamus activations was found when receiving fair offers from proposers in high economic status rather than ones in low economic status. Moreover, participants rejected more unfair offers, and stronger right MPFC activations were observed during acceptance of unfair offers by individuals in high economic status compared with ones in low economic status.

### Modulation of Perception of Unfairness by Economic Status

Consistent with our prediction, lower fairness ratings were revealed in the *High* economic status condition than in the *Low* economic status condition, whether participants received unfair or fair offers. As proposers in high economic status owned more resources than participants in low economic status, the lower ratings suggested that people in superior economic status were expected to give higher offers.

Significant activation of thalamus was identified in interaction between economic status and perception of unfairness of responders. Activations to fair offers were higher in the *High* economic status condition compared with those in the low condition. Previous studies about processing of social status revealed that the thalamus was most activated when viewing information from superior status, and it was argued by the researchers that thalamus was associated with an emotional arousal response to the superior player (Zink et al., 2008). Since economic status and social status were both involved in human social hierarchies (Zink et al., 2008), it suggested that activation of thalamus in this study might also reflect such emotional arousal related to economic status. Moreover, as shown in fairness ratings to fair offers, participants gave lower ratings to offers in the *High* (relative to *Low*) economic status condition, indicating that people felt unfair even if proposers in high economic status gave equal splits. So the increasing activation of thalamus might be related to the emotional arousal so that participants might feel higher level of unfairness to proposers in high economic status, even when they proposed fair offers. Additionally, consistent with previous studies in which thalamus engaged in processing of perception of unfairness (Kirk et al., 2011; Servaas et al., 2015), stronger activations to unfair offers were observed both in the *High* and *Low* economic status condition.

### Modulation of Responses during Unfair Trials by Economic Status

Participants were more likely to reject unfair offers when the proposers were in high economic status rather than in low economic status, indicating that economic status exerted influence on response to unfair offers. Furthermore, longest RTs were found when participants were offered ¥20 from proposers in high economic status. But if offered by proposers in low economic status, the longest RTs of responders were observed when the offer was ¥15. This result indicated that participants had to make tradeoff between self-interest and fairness perception at a higher offer when offered by proposers in high economic status than in low economic status. In consideration of the higher expectation on distribution from proposers in high economic status, responses to proposers in high economic status became a time-consuming work at a lower unfairness level than responses to ones in low economic status.

Activation of MPFC revealed higher activations to accept unfair offers from proposers in high economic status compared with in low economic status. MPFC was engaged in processing of socioeconomic status and associated with recognizing intentions and motives of other people (Frith and Frith, 2003; Frith and Frith, 2006; Zink et al., 2008). Although unfair offers from people in high economic status were more likely to be rejected and rated as more unfair, however, more rejections leaded to a higher cost of participants. Higher activations in MPFC suggested that, in view of personal loss, participants would accept the unfair offers from proposers in high economic status after thinking about the thoughts and feelings of them in order to rationalize the decisions from proposers.

## Conclusion

The present study explored how economic status would modulate unfairness-related decision making. Focusing on modulation of perception of unfairness by economic status, fairness ratings were found lower when offered by proposers in high economic status than in low economic status, and stronger thalamus activations were induced when receiving fair offers from proposers in high economic status than in low economic status. With respect to modulation of responses to unfairness by economic status, responses to proposers in high (relative to low) economic status became most time-consuming at a lower unfairness level, and people tended to reject unfair offers from proposers in high economic status. Moreover, increased MPFC activations were observed when accepting unfair offers from individuals in high economic status. To conclude, both perception of unfairness and responses to it were behaviorally and neurally modulated by economic status.

## Ethics Statement

This study was carried out in accordance with the recommendations of the Ethics Committee of East China Normal University with written informed consent from all subjects. All subjects gave written informed consent in accordance with the Declaration of Helsinki. The protocol was approved by the Ethics Committee of East China Normal University.

## Author Contributions

YZ, XC, LZ, GY, and XG devised the concept and supervised the study. YZ and JX collected the data. YZ, XC, LZ, LL, and XG joined in the interpretation of data. YZ, XC, LZ, and LL carried out the writing of the manuscript.

## Conflict of Interest Statement

The authors declare that the research was conducted in the absence of any commercial or financial relationships that could be construed as a potential conflict of interest.
